# Accelerated cellular senescence in degenerate intervertebral discs: a possible role in the pathogenesis of intervertebral disc degeneration

**DOI:** 10.1186/ar2198

**Published:** 2007-05-11

**Authors:** Christine Lyn Le Maitre, Anthony John Freemont, Judith Alison Hoyland

**Affiliations:** 1 Tissue Injury and Repair Group, School of Medicine, Stopford Building, The University of Manchester, Oxford Road, Manchester, UK, M13 9PT

## Abstract

Current evidence implicates intervertebral disc degeneration as a major cause of low back pain, although its pathogenesis is poorly understood. Numerous characteristic features of disc degeneration mimic those seen during ageing but appear to occur at an accelerated rate. We hypothesised that this is due to accelerated cellular senescence, which causes fundamental changes in the ability of disc cells to maintain the intervertebral disc (IVD) matrix, thus leading to IVD degeneration. Cells isolated from non-degenerate and degenerate human tissue were assessed for mean telomere length, senescence-associated β-galactosidase (SA-β-gal), and replicative potential. Expression of *P16*^*INK*4*A *^(increased in cellular senescence) was also investigated in IVD tissue by means of immunohistochemistry. RNA from tissue and cultured cells was used for real-time polymerase chain reaction analysis for *matrix metalloproteinase-13*, *ADAMTS 5 *(a disintegrin and metalloprotease with thrombospondin motifs 5), and *P16*^*INK*4*A*^. Mean telomere length decreased with age in cells from non-degenerate tissue and also decreased with progressive stages of degeneration. In non-degenerate discs, there was an age-related increase in cellular expression of *P16*^*INK*4*A*^. Cells from degenerate discs (even from young patients) exhibited increased expression of *P16*^*INK*4*A*^, increased SA-β-gal staining, and a decrease in replicative potential. Importantly, there was a positive correlation between *P16*^*INK*4*A *^and matrix-degrading enzyme gene expression. Our findings indicate that disc cell senescence occurs *in vivo *and is accelerated in IVD degeneration. Furthermore, the senescent phenotype is associated with increased catabolism, implicating cellular senescence in the pathogenesis of IVD degeneration.

## Introduction

Approximately 11 million people in the UK experience low back pain (LBP) for at least 1 week each month, leading to a considerable loss of working days and impacting significantly on the National Health Service. The cause of LBP is not known, but it is the intervertebral disc (IVD) and the age-related degenerative changes that occur within it that have been most frequently associated with LBP [1]. The incidence of disc degeneration increases with age, and the majority of lumbar IVDs show some evidence of degeneration by the fifth decade [2]. Although imaging studies indicate a link between degeneration of the IVD and LBP [1], clearly not all degenerate discs are symptomatic. Discs from symptomatic and asymptomatic individuals show similar radiographic, structural, and biochemical features. However, people who have LBP exhibit more severe degeneration than those who are asymptomatic, suggesting that IVDs of symptomatic individuals undergo either an acceleration or exacerbation (possibly due to environmental or genetic factors) of the ageing process. Thus, disc degeneration can be viewed as a predictable natural part of ageing, which in some people occurs at an accelerated rate for reasons that are currently unknown.

During ageing and degeneration, the matrix of the IVD undergoes substantial structural, molecular, and mechanical changes, including a loss in the demarcation between the annulus fibrosus (AF) and the nucleus pulposus (NP), alterations in collagen content, and a decrease in proteoglycan, resulting in loss of structural integrity, decreased hydration, and an inability to withstand load [3,4]. Because matrix changes largely reflect alterations in the biology of the cells, it is not surprising to find that during ageing and degeneration, the cells of the NP exhibit altered patterns of gene and protein expression for matrix molecules, degrading enzymes, and catabolic cytokines [5-9]. Accompanying this is a deterioration in the overall function of the disc cells, together with a decrease in tissue cellularity and cell viability of remaining disc cells, leading to an age-related impairment of IVD repair [6].

Cellular processes that lead to a reduction in fully functional cells and altered cellular activity include apoptosis and cellular senescence. Although apoptosis has been reported in age-related IVD degeneration, with higher rates of apoptosis present in older individuals [10], no studies, to date, have comprehensively investigated cellular senescence in ageing or degenerate IVDs. The accumulation of senescent cells *in vivo *with age, together with their changed pattern of gene expression [11], implicates cellular senescence in ageing and age-related pathologies. Indeed, Roberts and colleagues [12] and Gruber and colleagues [13] have shown increased staining for senescence-associated β-galactosidase (SA-β-gal) in cells from herniated discs and degenerate discs, respectively. Based on this one biomarker of senescence, they postulate that cellular senescence may be involved in the pathogenesis of disc degeneration. Similarly, the involvement of cellular senescence has been linked to osteoarthritis, and investigators have shown that chondrocytes in articular cartilage from older individuals and osteoarthritic cartilage display a senescent phenotype (as assessed by several markers) that correlates with changes in matrix homeostasis, leading to matrix destruction [14,15]. However, to date, no such studies correlating senescence and altered cell function have been conducted in cells from degenerate IVD tissue.

Here, we hypothesise that cellular senescence (assessed by mean telomere length [MTL], SA-β-gal staining, *p16*^*INK*4*A *^expression, and cell growth kinetics) occurs at an accelerated rate in IVD degeneration and that, importantly, the senescent phenotype is related to altered disc cell function associated with the characteristic features of IVD degeneration.

## Materials and methods

### Tissue samples

Human IVD tissue was obtained either at surgery, where patients were selected on the basis of magnetic resonance imaging-diagnosed degeneration and progression to anterior resection for either spinal fusion or disc replacement surgery for chronic LBP, or at post mortem examination. Whole discs were removed (as detailed previously [9]) following local research ethics committee approval and informed consent of the patient or relatives. Herniated disc samples were excluded from the study.

#### General procedure for tissue specimens

A block of tissue (incorporating AF and NP in continuity) was fixed in 10% neutral buffered formalin and processed to paraffin wax. Sections were taken for haematoxylin and eosin staining to score the degree of morphological degeneration according to previously published criteria [8]. In brief, sections were scored for the presence of cell clusters and fissures and for loss of demarcation and haematoxophilia (indicating reduced proteoglycan content). A score of 0 to 3 indicates a histologically normal (non-degenerate) IVD, 4 to 7 indicates evidence of intermediate degeneration, and 8 to 12 indicates severe degeneration. Additional sections were taken for immunohistochemistry (IHC).

### Isolation of disc cells

Whole disc tissue was separated into NP and AF and finely minced and digested with 2 U/ml protease (Sigma-Aldrich Company Ltd., Poole, UK) in Dulbecco's modified Eagle's medium (DMEM) + F-12 media for 30 minutes at 37°C and washed twice in DMEM + F-12. NP and AF cells were isolated in 2 mg/ml collagenase type 1 (Invitrogen Corporation, Paisley, UK) for 4 hours at 37°C.

### Evidence for senescence biomarkers *in vivo*

#### Telomere length assay

Following extraction of cells from IVD tissue, 31 disc cell samples (samples 1 to 31 inclusive in Table 1) were taken for DNA extraction and analysis of MTL. Genomic DNA (gDNA) was isolated from approximately 1 × 10^6 ^cells by means of a DNeasy kit (Qiagen Ltd., Crawley, West Sussex, UK) according to the manufacturer's instructions. Analysis of MTL was performed using the Telo TTAGGG telomere length assay according to the manufacturer's instructions (Roche Diagnostics Ltd, Burgess Hill, UK). Briefly, 1 μg of gDNA was digested with Hinf I and Rsa I for 2 hours and separated by electrophoresis. Southern transfer was performed and terminal restriction fragments were detected by hybridization to a digoxigenin-labeled telomeric oligonucleotide and chemiluminescence detection by alkaline phosphatase-conjugated anti-digoxigenin antibodies according to the manufacturer's protocol. Membranes were exposed to x-ray film for 5 minutes, and the MTL was determined using Gene Snap and Gene Tools from Syngene (SLS, Manchester, UK). Regression analysis and Spearman rank correlation were performed to analyse correlations between age and MTL in non-degenerate and degenerate discs. Multivariate linear regression adjusted for age (using Stata 9 statistical package; StataCorp LP, College Station, TX, USA) was used to assess the association between MTL and IVD degeneration. Mann-Whitney *U *tests were used to investigate statistical differences in MTL with degree of degeneration.

**Table 1:** Intervertebral disc samples used for telomere length assay, senescence-associated β-galactosidase staining, and *p16*^*INK*4*A *^immunohistochemistry

Laboratory number	Gender	Age (years)	Cell type	Disc level	Cell source	Histological grade
1	M	37	AF	L4/5	PM	1
2	M	37	AF	L5/S1	PM	1
3^a^	M	37	NP	L4/5	PM	1
4^a^	M	37	NP	L5/S1	PM	1
5	M	47	AF	L2/3	PM	1
6	M	47	AF	L3/4	PM	1
7^a^	M	47	NP	L2/3	PM	1
8	M	47	NP	L3/4	PM	1
9	M	47	NP	L5/S1	PM	1
10	M	47	AF	L4/5	PM	2
11	M	47	NP	L4/5	PM	2
12	M	59	NP	L4/5	PM	2
13	M	59	AF	L4/5	PM	2
14	M	62	AF	L3/4	PM	2
15	M	62	AF	L4/5	PM	2
16^a^	M	62	NP	L3/4	PM	2
17	M	62	NP	L4/5	PM	2
18	M	37	AF	L1/2	PM	3
19	M	37	NP	L1/2	PM	3
20	M	74	AF	L3/4	PM	3
21	M	37	AF	L2/3	PM	4
22	M	37	AF	L3/4	PM	4
23	M	37	NP	L2/3	PM	4
24	M	37	NP	L3/4	PM	4
25^a^	F	49	NP	L4/5	Surgical	4
26	M	44	NP	L4/5	Surgical	5
27	M	62	AF	L5/S1	PM	5
28	M	62	NP	L5/S1	PM	5
29	M	74	AF	L4/5	PM	5
30	M	74	AF	L2/3	PM	6
31^a^	F	49	NP	L5/S1	Surgical	8
32	F	15	Tissue	L4/5	Surgical	0
33	F	27	Tissue	L5/S1	Surgical	0
34	M	39	Tissue	L4/5	Surgical	0
35	F	44	Tissue	L4/5	Surgical	0
36	F	20	Tissue	L5/S1	Surgical	2
37	M	40	Tissue	L4/5	Surgical	2
38	M	47	Tissue	L4/5	Surgical	2
39	F	27	Tissue	L4/5	Surgical	3
40	M	31	Tissue	L4/5	Surgical	3
41	F	57	Tissue	L4/5	Surgical	3
42	M	59	Tissue	L5/S1	Surgical	3
43	M	28	Tissue	L4/5	Surgical	5
44	F	34	Tissue	L3/4	Surgical	5
45	M	39	Tissue	L5/S1	Surgical	5
46	M	55	Tissue	L3/4	Surgical	5
47	F	27	Tissue	L4/5	Surgical	7
48	F	56	Tissue	L5/S1	Surgical	7
49	M	33	Tissue	L5/S1	Surgical	8
50	F	40	Tissue	L4/5	Surgical	8
51	M	54	Tissue	L4/5	Surgical	8
52	M	32	Tissue	L4/5	Surgical	10
53	F	41	Tissue	L5/S1	Surgical	12

Intervertebral disc samples 1 to 31 were used for telomere length assay, and samples 32 to 53 were used for *p16*^*INK*4a ^immunohistochemistry. ^a^Intervertebral disc samples used for senescence-associated β-galactosidase staining. AF, annulus fibrosus; F, female; M, male; NP, nucleus pulposus; PM, postmortem tissue.

#### Expression and localisation of *P16*^*INK*4*A*^

IHC was used to localise the senescence marker *P16*^*INK*4a ^in 22 paraffin-embedded disc samples (samples 32 to 53 in Table 1). Tonsil tissue was used as a positive control. The IHC protocol followed was as previously published [5]. Briefly, following blocking of endogenous peroxidase and antigen retrieval with citrate buffer at 95°C for 20 minutes, sections were incubated overnight at 4°C with mouse monoclonal primary antibody against human *p16*^*INK*4a ^(Autogen Bioclear UK Ltd., Calne, Wiltshire, UK) (1:300 dilution in 25% wt/vol rabbit serum in 1% wt/vol bovine serum albumin [Sigma-Aldrich Company Ltd.]). Negative controls in which mouse immunoglobulin G (IgG) (Dako UK Ltd., Ely, Cambridgeshire, UK) replaced the primary antibody were used. After washing, sections were incubated with biotinylated rabbit anti-mouse antiserum (1:400; Dako UK Ltd.) for 30 minutes at room temperature. Disclosure of secondary antibody binding was by the streptavidin-biotin complex (Dako UK Ltd.) technique with 3,3'-diaminobenzidine tetrahydrochloride solution (Sigma-Aldrich Company Ltd.). Sections were counterstained with Mayers Haematoxylin (Raymond A Lamb Limited, Eastbourne, East Sussex, UK), dehydrated, and mounted in XAM (BDH, Liverpool, UK).

For analysis, each disc section was divided morphologically into three areas: the NP, inner AF (IAF), and outer AF (OAF). Regions situated at the junction of IAF and OAF or of NP and IAF were not included in the analysis. Within each area, five fields of view were analysed and the percentage immunopositivity was calculated. Data were non-parametric, thus Mann-Whitney *U *tests were used to compare the numbers of immunopositive cells in degenerate groups (4 to 7 and 8 to 12) to non-degenerate discs (scores 0 to 3) for each area of the disc. Regression analysis and Spearman rank correlation were also performed to analyse correlations between age and *p16*^*INK*4a ^immunopositivity. In addition, multivariate linear regression adjusting for age was performed to analyse correlations between grade of degeneration and *p16*^*INK*4a ^immunopositivity.

#### Senescence-associated β-galactosidase staining

Following extraction of cells from IVD tissue, six samples of NP cells (Table 1) were taken for SA-β-gal staining. Directly extracted cells were seeded onto 10-cm^2 ^flaskettes (SLS) at a cell density of 0.2 × 10^6 ^cells per flaskette. Cells were cultured in standard media [9] on flaskettes for 48 hours and then fixed in 4% wt/vol paraformaldehdye/phosphate-buffered saline (PBS) for 20 minutes. Following washing in PBS, cells were stained overnight for SA-β-gal using the β-Gal Staining Set (Roche Diagnostics Ltd), with buffer adjusted to pH 6. Sections were washed in PBS, counterstained with Mayers Haematoxylin (Raymond A Lamb Ltd), dehydrated, and mounted in XAM (BDH). Cells were visualised using a Leica RMDB research microscope (Leica Camera Limited, Knowlhill, Milton Keynes, UK), images were captured using a digital camera and Bioquant Nova image analysis system (Bioquant Image Analysis Corporation, Nashville, TN, USA), and the percentage of SA-β-gal-positive cells was calculated.

### Senescence biomarkers in human intervertebral disc cells *in vitro*

#### Assessment of growth kinetics

Growth kinetics were examined in NP cells extracted from four discs (two non-degenerate discs from one post mortem [L2/3: grade 1, L4/5: grade 2; 37-year-old male] and two degenerate discs from one patient undergoing surgery [L4/5: grade 4, L5/S1: grade 8; 49-year-old male]). Following extraction, cells were seeded into T75 flasks at a cell density of 0.25 × 10^6^, cultured to 75% confluence, and serially passaged until cells ceased dividing (failure of population doubling in 4 weeks). Time in culture and cell number were recorded for each passage, and cumulative population doublings were calculated.

At each passage, an aliquot of approximately 1 × 10^6 ^cells was taken for analysis of MTL, and regression analysis and Spearman rank correlation were performed to analyse MTL in cells following prolonged culture. Aliquots of cells (0.5 × 10^6 ^cells) were also taken in duplicate prior to culture (that is, directly extracted cells) and at each passage for analysis of *p16*^*INK*4a^, *MMP-13 *(matrix metalloproteinase-13), *ADAMTS 5 *(a disintegrin and metalloprotease with thrombospondin motifs 5), and *hTERT *(human telomerase reverse transcriptase) gene expression.

#### Human telomerase reverse transcriptase polymerase chain reaction

Reverse transcriptase-polymerase chain reaction (PCR) was used to investigate the gene expression of *hTERT *in the samples detailed above to assess the ability of disc cells to repair telomeres and prevent telomere shortening. RNA was extracted with Trizol^® ^reagent (Invitrogen Corporation) and cDNA was synthesised using Bioscript RNase H minus reverse transcriptase (Bioline Ltd., London, UK) and random hexamers (Roche). PCR was performed with 5 μl of cDNA (50 ng/μl) from each test sample and positive control cDNA (generated from hTERT-infected cells (a kind gift from Basem Abdallah and Moustapha Kassem, Odense University Hospital, Odense, Denmark)). Glyceraldehyde-3-phosphate dehydrogenase (*GAPDH*) primers were designed using Amplify 1.2 software (Professor B Engels, University of Wisconsin, USA) and gene specificity was confirmed by Basic Local Alignment Search Tool (BLAST) searches (GenBank database sequences). *hTERT *primers were a kind gift from B. Abdallah and M. Kassem (Table 2).

**Table 2:** Polymerase chain reaction primer and probe sequences, amplicon sizes, and efficiencies

Standard polymerase chain reaction conditions				
Target	Forward primer	Reverse primer		Amplicon size
GAPDH	5' CCC ATC ACC ATC TTC CAG G 3'	5' GGC CAT CCA CAG TCT TCT G 3'		354 bp
hTERT	5' GCC TGA GCT GTA CTT TGT CAA 3'	5' AGG CTG CAG AGC AGC GTG GAG AGG 3'		422 bp
Real-time polymerase chain reaction primers and probes				

Target	Forward primer	Probe	Reverse primer	Efficiency

18s	PDAR	PDAR	PDAR	99.65%
P16^*INK*4a^	5' GGC TCT ACA CAA GCT TCC TTT CC 3'	5' 6 FAM – CCC CCA CCC TGG CTC TGA CCA – TAMRA	5' TCA TGA CCT GCC AGA GAG AAC A 3'	99.22%
MMP-13	5' CCC CAG GCA TCA CCA TTC AAG 3'	5' 6 FAM – AGG GGT CCT GGC TGC CTT CCT CTT C – TAMRA 3'	5' GAC AAA TCA TCT TCA TCA CCA CCA C 3'	99.77%
ADAMTS 5	5' GGA CCT ACC ACG AAA GCA GAT C 3'	5' 6 FAM – CCC AGG ACA GAC CTA CGA TGC CAC C – TAMRA 3'	5' GCC GGG ACA CAC GGA GTA 3'	99.74%

ADAMTS 5, a disintegrin and metalloprotease with thrombospondin motifs 5; bp, base pairs; GAPDH, glyceraldehyde-3-phosphate dehydrogenase; hTERT, human telomerase reverse transcriptase; MMP-13, matrix metalloproteinase-13; PDAR, pre-developed assay reagent.

### Correlation of senescent phenotype with altered expression of matrix-degrading enzyme genes

Real-time PCR was performed for *18s*, *p16*^*INK*4a^, *MMP-13*, and *ADAMTS 5 *using the standard curve method of analysis on directly extracted cells and expanded cells.

#### Primers and probe design

Primers and probes were designed using the Primer Express program (Applied Biosystems, Warrington, UK) within a single exon to allow detection of target genes in gDNA and cDNA samples. Total gene specificity was confirmed by BLAST searches (GenBank database sequences). Primers and probes were purchased from Applied Biosystems (Table 2).

#### Genomic standard curve

gDNA was used to generate standard curves for absolute quantification of copy number per reaction. Briefly, gDNA (Promega UK Ltd., Southampton, UK) was homogenised, diluted to 25,000 pg/μl, and sonicated (Soniprep 150; MSE, Wolf Laboratories Limited, Pocklington York, UK) on ice. Serial dilutions of gDNA were prepared to generate standards with gene copy numbers of 15,000, 3,000, 600, 120, 24, and 0 copies per 2 μl of gDNA.

#### Polymerase chain reaction amplification

PCRs were performed and monitored using the ABI Prism 7000 Sequence detection System (Applied Biosystems). The PCR master mix was based on the AmpliTaq Gold DNA polymerase (Applied Biosystems). On each real-time PCR plate, a gDNA standard curve was included and cDNA samples (2 μl [50 ng cDNA/μl] in a total volume of 25 μl) were analysed in duplicate. Primers were used at a concentration of 900 nM, and probe at a concentration of 250 nM. After an initial denaturation step and Taq activation at 95°C for 10 minutes, the cDNA products were amplified with 40 PCR cycles consisting of a denaturation step at 95°C for 15 seconds and an annealing and extension step at 60°C for 1 minute.

#### Analysis of real-time polymerase chain reaction

Following real-time amplification, the ABI Prism 7000 expressed the data as an amplification plot, from which a baseline was set from cycle number 3 up to a few cycles prior to the first visible amplification. A threshold was set at a level above background levels and within the exponential phase of the PCR amplification. Vales of Ct (cycle at which the set threshold is reached) were then exported into an Excel file (Microsoft Corporation, Manchester, UK), and absolute quantification analysis was performed using the gDNA standard curve.

#### Absolute quantification

Standard curves were generated for the housekeeping gene (*18s*) and each target gene by plotting log_10 _copy number against Ct value. Line of best fit was then drawn, and the equation of the line and R^2 ^was taken. Efficiency (E) was measured as E = 10 ^[-1/slope]^[16], R^2 ^values were accepted if greater than 0.95, and all efficiencies were 97% or greater (Table 2). Ct values for test samples were converted into copy number per 100 ng of cDNA using the appropriate standard curve for each gene. Copy numbers obtained for *18s *were used to generate a correction factor for normalization of target genes using the equation: (maximum *18s *copy number)/(*18s *copy number for each individual sample), and the correction factor was then multiplied by the copy number for each target gene for each sample to give copy number of target gene normalized to *18s *per 100 ng of cDNA. Regression analysis and Spearman rank correlation were performed to analyse correlations between *p16*^*INK*4a ^and matrix-degrading enzymes (*MMP-13 *and *ADAMTS 5*) gene expression.

## Results

### Evidence for senescence biomarkers *in vivo*

#### Mean telomere length in cells directly extracted from human intervertebral disc tissue

**Figure 1:**
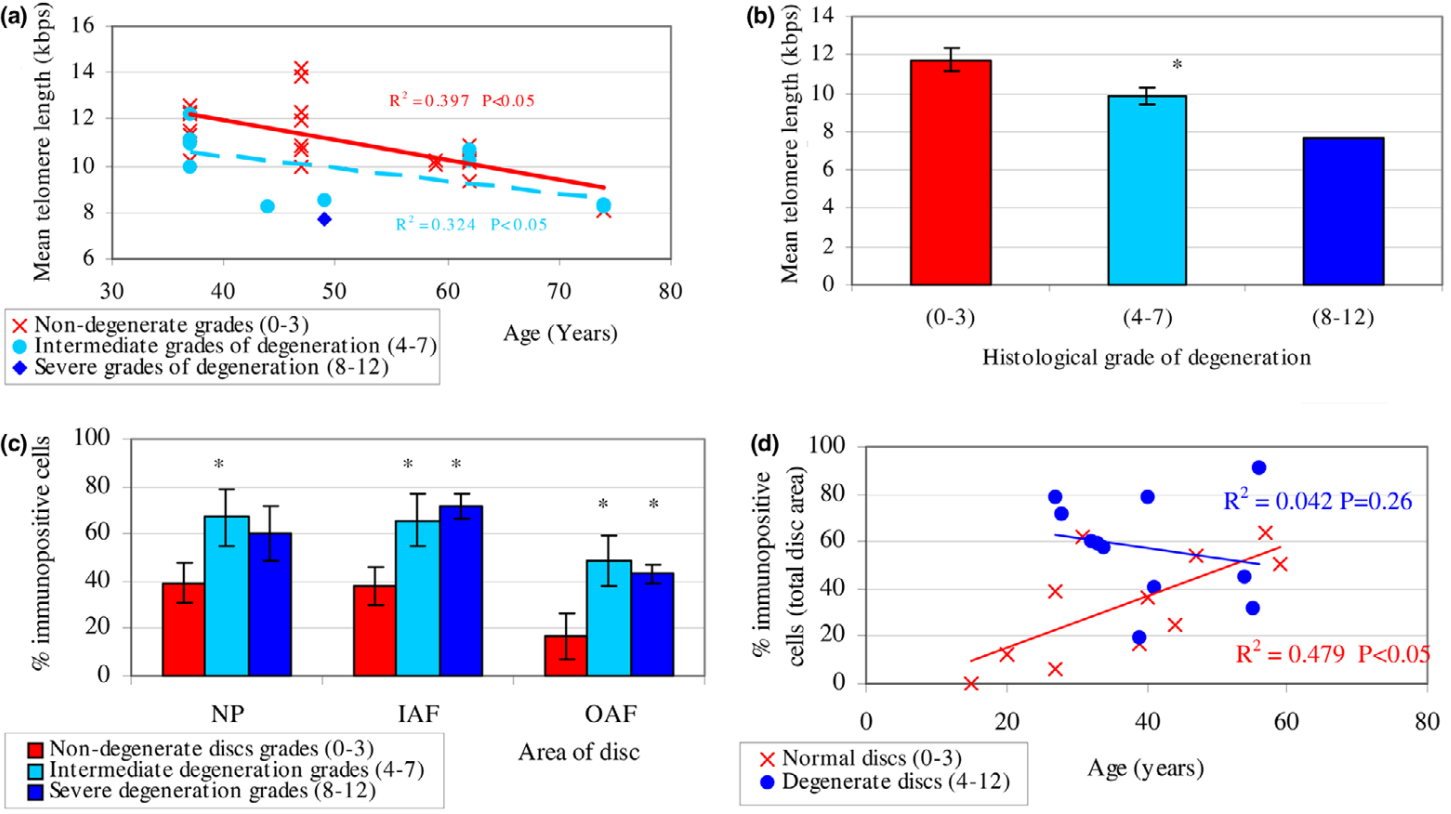
The expression of senescence biomarkers *in vivo*. **(a) **Mean telomere length (MTL) in cells directly extracted from non-degenerate and degenerate human intervertebral discs (IVDs): correlation with age. Samples are from 20 non-degenerate discs (6 aged 37 years, 7 aged 47 years, 2 aged 59 years, 4 aged 62 years, and 1 aged 74 years), 10 intermediate degenerate discs (4 aged 37 years, 1 aged 44 years, 1 aged 49 years, 2 aged 62 years, and 2 aged 74 years), and 1 severely degenerate disc (aged 49 years). Spearman rank correlation *P *< 0.05. **(b) **MTL in cells directly extracted from non-degenerate and degenerate human IVDs: effect of degree of degeneration. *Intermediate degenerate samples are significantly different from non-degenerate samples (*P *< 0.05). Disc samples are as described in **(a)**. Data are shown as average MTL ± standard error of the mean (SEM) for each disease state. **(c) **Quantification and localisation of *p16*^*INK*4a ^immunopositivity in human IVDs correlated with degree of degeneration. *Samples are significantly different from non-degenerate samples (*P *< 0.05). Samples are from 11 non-degenerate discs, 6 intermediate degenerate discs, and 5 severely degenerate discs. Averages ± SEM are presented. **(d) ***p16*^*INK*4a ^immunopositive cells in human IVDs correlated with age. Samples are as detailed in **(c)**. Intermediate degenerate (grades 4 to 7) and severely degenerate (grades 8 to 12) samples are grouped for correlation analysis. Spearman rank correlation for non-degenerate samples *P *< 0.05 and for degenerate samples *P *= 0.26. IAF, inner annulus fibrosus; kbp, kilobase pairs; NP, nucleus pulposus; OAF, outer annulus fibrosus.

MTL was investigated in cells directly extracted from 20 histologically non-degenerate discs, 10 histologically graded intermediate degenerate discs, and 1 histologically graded severe degenerate disc. MTL decreased significantly with increasing age in non-degenerate and degenerate discs (*P *< 0.05), with an average decrease in MTL of 0.85 kbp per decade of life in non-degenerate discs (Figure 1a). Interestingly, the MTL differed according to the degree of degeneration in two discs from the same individual (grade 4 disc: MTL 8.56; grade 8 disc: MTL 7.7), and following the statistical correction of results for age, a significant correlation was observed between degeneration state (that is, non-degenerate versus degenerate) and MTL (*P *< 0.05). Degenerate discs (grades 4 to 7) showed significantly shorter MTL compared to non-degenerate discs (*P *< 0.05), with a progressive shortening seen with increasing grade of degeneration (Figure 1b).

#### *p16*^*INK*4*A *^Immunohistochemical localisation in human intervertebral disc tissue

**Figure 2:**
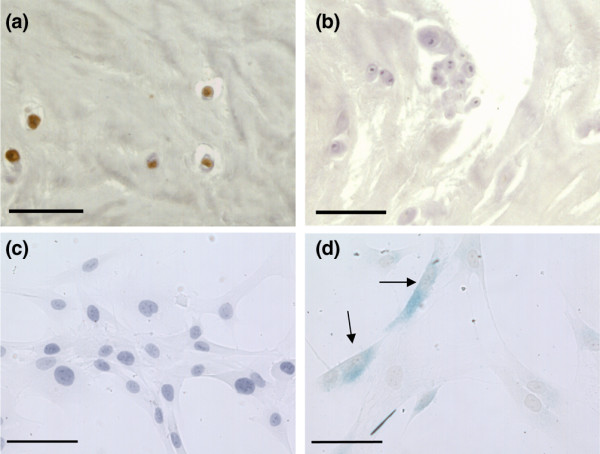
Senescence biomarker immunohistochemistry. **(a) ***p16*^*INK*4a ^immunopositivity in the nucleus pulposus of human intervertebral discs. **(b) **Immunoglobulin G controls were negative. **(c) **Senescence-associated β-galactosidase staining in directly extracted cells from non-degenerate discs. **(d) **Senescence-associated β-galactosidase staining in directly extracted cells from degenerate discs (positive cells indicated with arrows). Scale bars = 190 μm **(a, b) **and 370 μm **(c, d)**.

Immunopositive cells were found in all areas of the disc, although less positivity was observed in the OAF (Figure 1c). Degenerate discs showed significantly higher proportions of *p16*^*INK*4a ^immunopositive cells than non-degenerate discs in all areas of the IVD (*P *< 0.05), except for the NP in severe grades (8 to 12) of degeneration (Figure 1c), where there was a non-significant increase compared to non-degenerate NP. Non-degenerate disc samples showed a significant positive correlation in *p16*^*INK*4a ^immunopositive cells with increasing age (*P *< 0.05), although in degenerate samples no such correlation was observed (*P *= 0.26) (Figure 1d). A significant positive correlation was observed between grade of degeneration and number of *p16*^*INK*4a ^immunopositive cells following correction for age (*P *< 0.05). Immunoreactivity for *p16*^*INK*4a ^was restricted to the nucleus and cytoplasm of native disc cells in all disc samples investigated, with no immunopositivity observed in the matrix or blood vessels (Figure 2a, b). IgG controls were all negative.

#### Senescence-associated β-galactosidase staining in cells directly extracted from human intervertebral disc tissue

SA-β-gal staining was not observed in any of the NP cells isolated from the four non-degenerate discs investigated. However, staining was observed in a number of NP cells extracted from both grade 4 (12.25% SA-β-gal-positive) and grade 8 (10.25% SA-β-gal-positive) degenerate discs (Figure 2c, d).

### Senescence biomarkers in human intervertebral disc cells *in vitro*

**Figure 3:**
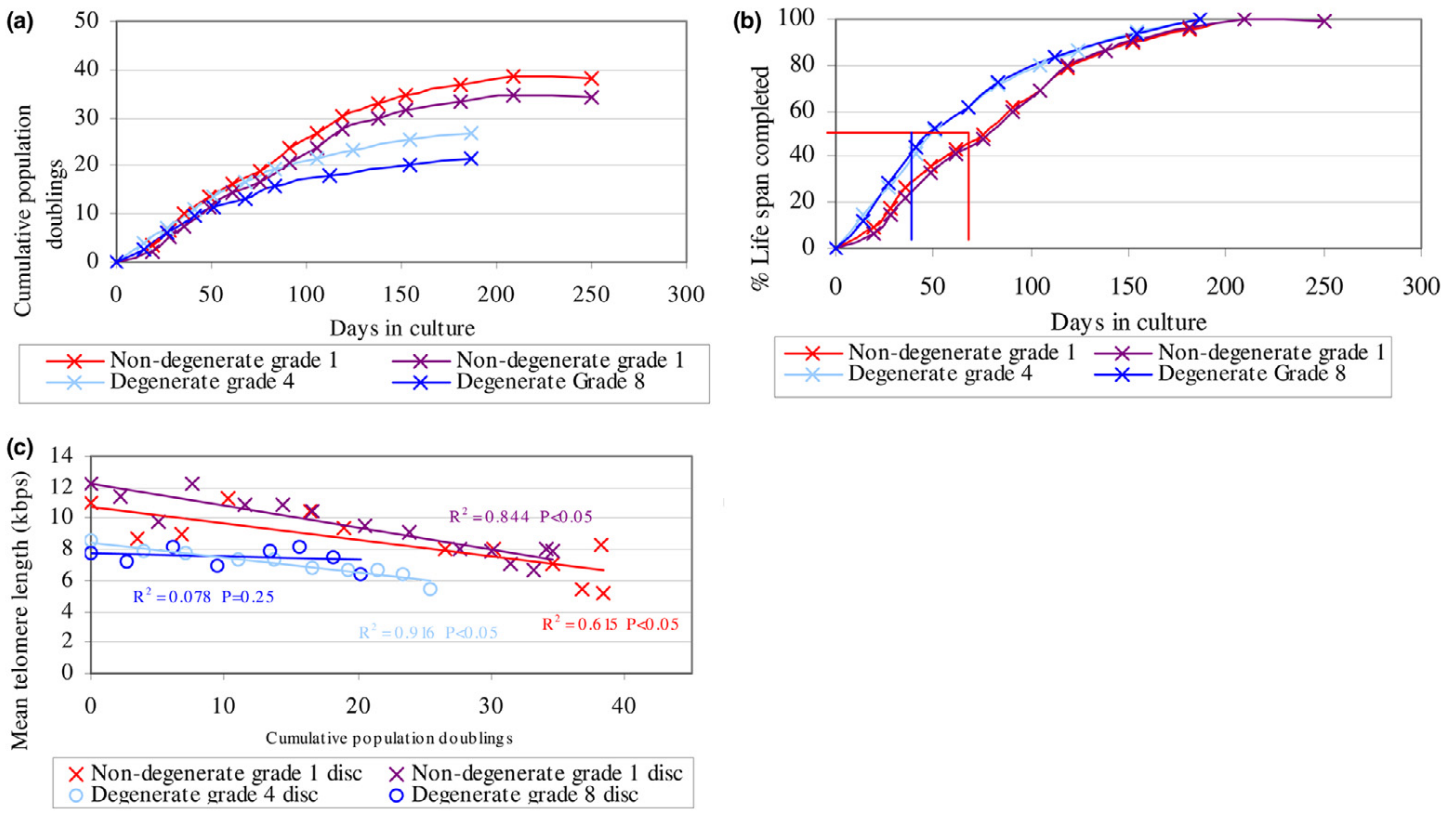
Senescence biomarkers in human intervertebral disc (IVD) cells *in vitro*. **(a) **Cell growth kinetics: cumulative population doublings in nucleus pulposus (NP) cells extracted from non-degenerate and degenerate IVDs. **(b) **Percentage of life span completed over time in culture of NP cells extracted from non-degenerate and degenerate IVDs. **(c) **Mean telomere length in NP cells extracted from non-degenerate and degenerate IVDs with increasing population doubling. Samples used consisted of two non-degenerate discs from one post mortem (L2/3: grade 1, L4/5: grade 2; 37-year-old male) and two degenerate discs from one patient undergoing surgery (L4/5: grade 4, L5/S1: grade 8; 49-year-old male).

Culture of NP cells derived from two non-degenerate discs showed similar growth kinetics, achieving 34 and 38 cumulative population doublings before reaching senescence (Figure 3a). NP cells derived from degenerate discs showed slower growth kinetics with a reduced capacity to proliferate, achieving replicative senescence (RS) after 27 cumulative population doublings (cells from a grade 4 disc) and 21 cumulative population doublings (cells from a grade 8 disc) (Figure 3a). Cells derived from degenerate NP completed 50% of their life span within 50 days in culture, whereas cells derived from non-degenerate NP were cultured for approximately 75 days prior to 50% of their life span being completed (Figure 3b).

MTL in NP cells derived from non-degenerate discs showed a negative correlation with increasing population doublings (*P *< 0.05) (Figure 3c), with telomere shortening of 180 to 210 base pairs (bp) per cell division (Figure 3c). A negative correlation was also seen in the NP cells from the low-grade degenerate disc (*P *< 0.05) but not in the NP cells from the severe degenerate disc (*P *= 0.25) (Figure 3c).

### Expression of human telomerase reverse transcriptase by intervertebral disc cells

GAPDH was expressed in all samples, but hTERT was detected only in the positive control, with no expression seen in any of the disc samples.

### Correlation of senescence phenotype with features of disc degeneration

#### Evidence from directly extracted cells

No gene expression for *p16*^*INK*4a^, *MMP-13*, or *ADAMTS 5 *was observed in directly extracted NP cells from non-degenerate discs, but expression for these genes was seen in NP cells directly extracted from degenerate discs (average: *p16*^*INK*4a^, 1,893 copies/100 ng of cDNA; *MMP-13*, 9,386 copies/100 ng of cDNA; *ADAMTS 5*, 21,220 copies/100 ng of cDNA).

### Correlation of *p16*^*INK*4*A *^and matrix-degrading enzyme gene expression

**Figure 4:**
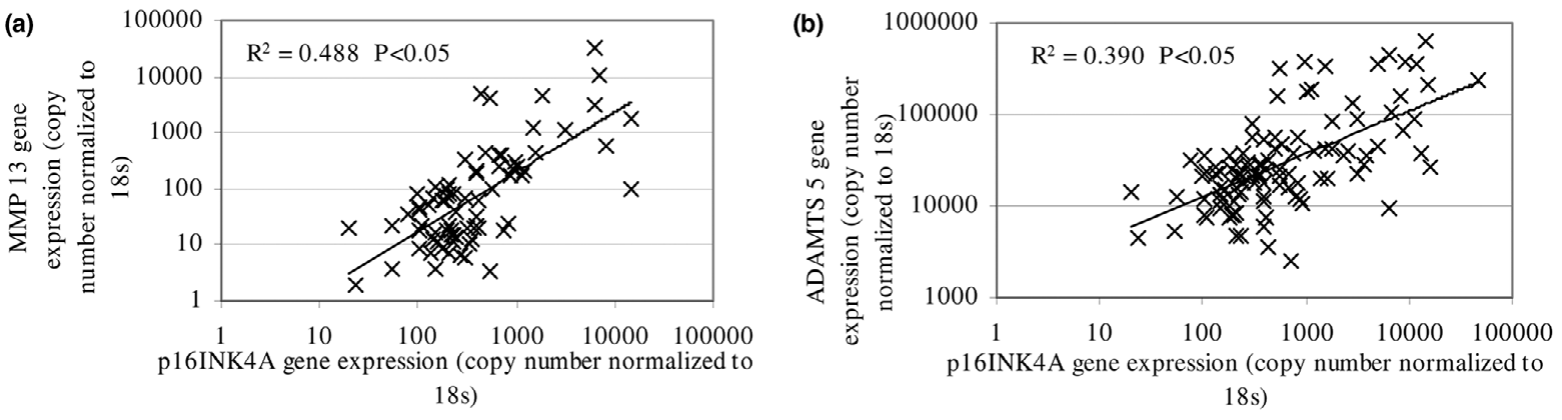
Correlation of senescent phenotype with expression of matrix-degrading enzymes. **(a) **Correlation of *MMP-13 *and *p16*^*INK*4a ^gene expression in human intervertebral disc (IVD) cells. Spearman rank correlation *P *< 0.05. **(b) **Correlation of *ADAMTS 5 *and *p16*^*INK*4a ^gene expression in human IVD cells. Spearman rank correlation *P *< 0.05. ADAMTS 5, a disintegrin and metalloprotease with thrombospondin motifs 5; MMP-13, matrix metalloproteinase-13.

The combination of all samples investigated demonstrated a significant positive correlation between *p16*^*INK*4a ^gene expression and the gene expression for the matrix-degrading enzymes *MMP-13 *and *ADAMTS 5 *(*P *values < 0.05) (Figure 4a, b).

## Discussion

We hypothesised that, during ageing and degeneration of the disc, the chondrocyte-like disc cells become senescent, resulting in phenotypic changes that can lead to the altered cell function and extracellular matrix characteristic of disc degeneration. This study has shown for the first time that in non-degenerate discs the incidence of senescent cells increases with age. In particular, we have found that telomeric erosion increases with age together with increased levels of *p16*^*INK*4a^. Importantly, this study has shown that degenerate discs exhibit accelerated senescence with decreased telomere length, reduced cell replication potential, and elevated levels of *p16*^*INK*4a ^and SA-β-gal staining compared to non-degenerate discs from age-matched individuals. Furthermore, the senescent phenotype is associated with features characteristic of disc degeneration, namely increased catabolic cell function.

There are two known mechanisms for the induction of senescence in a cell: RS and stress-induced premature senescence (SIPS). RS is generally regarded as the result of telomere shortening accumulated as cells undergo repeated cell divisions [17]. The exact turnover rate of NP cells in the IVD is not known but is thought to be low. However, Martin and Buckwalter [14] examined cells in articular cartilage, which share many characteristics with those of the NP, and suggested that although turnover is slow the very long life of the chondrocyte may mean that in older people chondrocytes may have gone through sufficient replications to induce RS. SIPS is the alternative explanation for cellular senescence and has come from the discovery that various insults, including mechanical load, high levels of oxygen and cytokines such as interleukin-1 (IL-1), can lead to cellular senescence [18,19]. This is an appealing explanation for the senescent biomarker expression seen in the degenerate IVD of young people as degeneration can be induced by mechanical load and cytokines such as IL-1, which we have shown to be increased in IVD degeneration [9]. Furthermore, the increased vascularisation also seen during disc degeneration [20,21] could lead to increased oxygen tension and hence induction of senescence.

One feature of senescent cells which appears as a universal and predictable marker is telomere shortening [22]. Telomeres are repetitive DNA sequences at the end of chromosomes which are essential for chromosomal replication but also help sustain normal chromosome function by sealing the chromosome ends and preventing enzymatic degradation. Upon each cell division, telomeres degrade because replication of the extreme ends of DNA is not possible. To counteract telomere shortening, cells can express the enzyme telomerase (hTERT), which synthesizes new telomeric repeats, thereby maintaining or increasing telomere length. We have demonstrated that the NP cells extracted from both the non-degenerate and degenerate IVD do not show expression of hTERT and thus are fully susceptible to telomere erosion.

Telomere length is often considered a good indicator of the cell's replicative history [17]. Telomeres, however, can also be shortened during SIPS in a manner independent from replication [18,23,24]. Thus, MTL can be considered a marker of replicative history and of the cumulative history of stress inducers of senescence, as well as an indicator of the probability of cell senescence [25]. In this study, we have investigated telomere erosion in disc cells both *in vitro *and *in vivo*. Martin and Buckwalter [14] demonstrated that *in vitro *telomeres in articular chondrocytes shortened by 100 to 200 bp per cell division, and Parsch and colleagues [26] showed telomere shortening of approximately 300 bp per cumulative population doubling in the same cell type. In the current study (albeit only in two samples), we demonstrated that in NP cells derived from non-degenerate discs, expansion in monolayer resulted in a progressive shortening of MTL, with a reduction of 180 to 210 bp per cellular division, matching the attrition rate seen *in vitro *in articular chondrocytes [14,26]. In NP cells isolated from the non-degenerate discs, RS was induced when telomeres reached a critical level of approximately 5 to 6.5 kbp, which matched the critical level of approximately 5 to 7.6 kbp observed previously in articular chondrocytes [14].

We have demonstrated that *in vivo *in 20 non-degenerate samples telomeres shortened at a rate of approximately 85 bp per year, suggesting an *in vivo *replication rate of one cell division every 2 years. The attrition rate seen in disc cells *in vivo *is higher than the 30 bp/year attrition rate seen in articular chondrocytes [27] but is similar to the attrition rate of 102 bp/year seen in iliac artery cells [28]. This would suggest that disc cells have a higher rate of cell turnover or are exposed to more stress than articular chondrocytes *in vivo*. Indeed, the degenerative process in IVD begins as early as the second decade of life, with associated increased occurrence of LBP [29]. However, articular cartilage does not show degenerative changes until later in life, with the incidence of osteoarthritis increasing dramatically after the age of 40 years [14]. Our data suggest that senescent cells accumulate in different tissues at different rates, with non-degenerate disc cells ageing faster than cells from articular cartilage, which may be a result of environmental factors such as mechanical stress, cytokine exposure, or injury. Furthermore, our data suggest that cells from degenerate discs exhibit accelerated senescence. (For example, the MTL of 7.7 kbp in a severe degenerate sample would have been predicted to be from an 80-year-old; however, this disc sample came from a donor who was only 49 years old.)

Hayflick [30] showed that normal cells could divide only a limited number of times in culture (the maximum number of divisions is known as the Hayflick limit), after which cells remain viable but are completely incapable of entering cell division and are thus termed senescent. Since this time, the reduced ability of cells to divide in culture has been used as an assessment of premature senescence [31]. The Hayflick limit for human fibroblasts has been estimated at approximately 60 population doublings, whereas the estimated limit for human chondrocytes is approximately 35 doublings [14]. We have shown that NP cells from non-degenerate discs were capable of 35 to 40 population doublings prior to reaching the Hayflick limit, which matches that seen previously for articular chondrocytes. However, in NP cells derived from degenerate discs, a reduced capability to divide was seen with cells capable of only 20 to 25 population doublings prior to senescence.

A number of studies have shown increased levels of *p16*^*INK*4a ^with increased occurrence of senescence [32,33]. *p16*^*INK*4a ^is thought to be involved in the activation of the retinoblastoma cell cycle inhibitory pathway, leading to permanent growth arrest and cellular senescence [34]. We have demonstrated that in non-degenerate discs *p16*^*INK*4a ^increases with age but that degenerate discs show overexpression of *p16*^*INK*4a ^compared to age-matched non-degenerate samples. This is similar to the increased expression of *p16*^*INK*4a ^seen in osteoarticular cartilage [35] and suggests that *p16*^*INK*4a ^may be physiologically involved in the senescence process, particularly as *p16*^*INK*4a ^may accumulate in response to specific forms of stress, including oxidative damage [18].

Since the initial description of the pH-dependent staining of senescent fibroblasts by β-galactosidase at pH 6 [36], this simple histological stain has been used in a number of studies to indicate the presence of senescent cells [14,27,37], including in the IVD [12,13]. Like Roberts and colleagues [12] and Gruber and colleagues [13], we have shown that NP cells stain for SA-β-gal, but our results differ in that we found no staining in non-degenerate NP cells. However, as in these previous studies, with degeneration, there was increased SA-β-gal staining. Because these discs (in our study) also showed shorter MTLs, reduced ability to divide, and increased numbers of *p16*^*INK*4a ^immunopositive cells compared to cells from non-degenerate discs, our data clearly illustrates an increase in cellular senescence in degenerate discs compared to non-degenerate discs, corroborating the recent data produced by Gruber and colleagues [13].

During cell senescence, cell function can deteriorate before cell cycle arrest occurs, with cells showing abnormal protein synthesis and an altered phenotype (including over expression of *p16*^*INK*4a ^[38]), and in chondrocytes, increased levels of MMPs and aggrecanases have been observed [37]. Here, we demonstrate for the first time that, in cells extracted from human NP tissue, increased levels of *p16*^*INK*4a ^were associated with increased gene expression of the degradative enzymes *MMP-13 *and *ADAMTS 5*, which is characteristic of disc degeneration [5,39]. We have previously shown that cells from degenerate discs respond differently to IL-1 compared to cells from non-degenerate discs [9]. Cellular senescence may be responsible for this as it has been shown that senescent cells show altered responses to cytokines and growth factors [15]. Our data indicate that the senescent phenotype is linked to the increased production of degradation enzymes which may be brought about by the catabolic cytokine IL-1 known to be increased in disc degeneration [9].

## Conclusion

We have shown tissue-specific cellular senescence and accelerated senescence in the degenerate IVD and that this is associated with increased catabolic cell function. Cellular senescence can be prevented, bypassed, or reversed in other settings and perhaps here too [35,40,41]. Our data suggest that disc cell senescence has an important role in the development and progression of IVD degeneration; thus, understanding the nature of cellular senescence will be paramount in devising new approaches for its prevention and treatment. Furthermore, the cellular senescence we have identified could be imperative in dictating the success of possible future biologic therapies, which may require the insertion of new metabolically active cells into the degenerate disc to achieve success.

## Abbreviations

ADAMTS 5 = a disintegrin and metalloprotease with thrombospondin motifs 5; AF = annulus fibrosus; BLAST = Basic Local Alignment Search Tool; bp = base pairs; Ct = cycle at which threshold is reached; DMEM = Dulbecco's modified Eagle's medium; GAPDH = glyceraldehyde-3-phosphate dehydrogenase; gDNA = genomic DNA; hTERT = human telomerase reverse transcriptase; IAF = inner annulus fibrosus; IgG = immunoglobulin G; IHC = immunohistochemistry; IL-1 = interleukin-1; IVD = intervertebral disc; LBP = low back pain; MMP-13 = matrix metalloproteinase-13; MTL = mean telomere length; NP = nucleus pulposus; OAF = outer annulus fibrosus; PBS = phosphate-buffered saline; PCR = polymerase chain reaction; RS = replicative senescence; SA-β-gal = senescence-associated β-galactosidase; SIPS = stress-induced premature senescence.

## Competing interests

The authors declare that they have no competing interests.

## Authors' contributions

CLM participated in the design of the study, performed the majority of the laboratory work and analysis, and drafted the manuscript. AJF participated in the design of the study and interpretation of data. JAH conceived the study, secured funding, contributed to the design and coordination of the study, and participated in interpretation of data and extensive preparation of the final manuscript. All authors read and approved the final manuscript.
